# Local Wound Infiltration for Thyroidectomized Patients in the Era of Multimodal Analgesia

**DOI:** 10.3390/medicina59091662

**Published:** 2023-09-14

**Authors:** Stiliani Laskou, Georgia Tsaousi, Chryssa Pourzitaki, Labrini Loukipoudi, Georgios Papazisis, Isaak Kesisoglou, Konstantinos Sapalidis

**Affiliations:** 1 3rd Surgical Department, School of Medicine, Aristotle University of Thessaloniki, 54124 Thessaloniki, Greece; 2 Clinic of Anesthesiology and Intensive Care, School of Medicine, Aristotle University of Thessaloniki, 54124 Thessaloniki, Greece; 3 Department of Clinical Pharmacology, Faculty of Medicine, School of Health Sciences, Aristotle University of Thessaloniki, 54006 Thessaloniki, Greece; chpour@gmail.com (C.P.);

**Keywords:** thyroidectomy, wound infiltration, postoperative analgesia

## Abstract

The first few hours following thyroidectomy are the most crucial for pain management. Adequate postoperative pain control, reduction in opioid abuse and the possibility of implementing one-day operations are the considered parameters when developing the postoperative analgesic strategy. A study of the available literature was conducted, exploring the efficacy of (open) thyroidectomy wound infiltration. Seventeen full-text RCTs were extracted. Local anesthetics and non-steroidal anti-inflammatory drugs were infiltrated. Emphasis was given to postoperative pain scores and requirements for rescue analgesia with opioids. Most authors agree that local wound infiltration for thyroidectomized patients is effective in the management of postoperative pain parameters. In the era of multimodal analgesia, thyroidectomy wound infiltration could represent an essential adjunct contributing to lower VAS scores and reduced opioid requirements.

## 1. Introduction

Surgical site infiltration represents an easy, safe and inexpensive analgesic method. The mechanism of action includes two pathways. The first includes the direct inhibition of noxious stimuli transmission on the wound surface by binding to fast sodium channels within the axon membrane interrupting the action potential. Moreover, it can reduce the local inflammatory response to injury by inhibiting the release of inflammatory mediators from neutrophils and neutrophil adhesion to the endothelium, as well as decreasing the formation of free oxygen radicals [1,2].

Wound infiltration has been mainly used for minor surgical procedures, while it seems to occupy an integral part in multimodal analgesia schemes for major procedures. As far as thyroid and parathyroid surgery is concerned, a few randomized controlled trials have tried to provide a comprehensive review utilizing various agents and protocols. In this narrative review, the efficacy of wound infiltration in the management of postoperative incision site pain after thyroidectomy is evaluated.

## 2. Material and Methods

A literature search using the terms “open thyroidectomy and/or parathyroidectomy and wound infiltration” was conducted in PubMed and Google Scholar, with a secondary search of the references of each included publication. Only local wound infiltration studies were included, excluding reviews referring to regional techniques such as cervical plexus block. Eligibility criteria were full text articles published in the English language, while all study designs were included.

Eighteen articles published between 1994 and April 2023 were identified [3,4,5,6,7,8,9,10,11,12,13,14,15,16,17,18,19]. Table 1 summarizes the reported trials. All of them were prospective, randomized trials except for one observational research based on a prior clinical trial [20]. Each article was carefully studied and a database was created, emphasizing the investigated preparation, the infiltration timing, the postoperative VAS score and the analgesic requirement, as well as the reduction in opioid consumption. 

## 3. Results

From the available data, most procedures were performed in adults (>16 years old). Apart from the fact that no difference regarding age between groups existed, no further age-subgroup analysis was conducted [3,4,5,6,10,11,16,17,18,19]. An ASA status of I or II was required when participating in the trial [5,8,9,10,13,14,16,17,18,19], although some authors include patients with a status of III [6,11,20]. Long-term analgesic, corticosteroid or sedative medication or the use of analgesics 7 days prior to the procedure was prohibited [7,9,10,11,13,14,15,16,17,18]. Three authors set as an exclusion criterion the prolongation of the procedure 11,15,16. A two-hour limit is set by Mismar et al. [15], while Sellami et al., expands this to 3 h [16]. The routine use of drainage is reported by only two authors [7,12].

Local anesthetics used for thyroidectomy wound infiltration include the amides bupivacaine, lidocaine and ropivacaine and the non steroidal anti-inflammatory drugs lornoxicam and diclofenac (NSAIDs). The majority of authors utilize bupivacaine 0.5% as an infiltrative agent. Seven authors compare bupivacaine 0.5% infiltration to the placebo, while one compares bupivacaine to diclofenac and one more compares between 5 and 10 mL of lidocaine 1%. Ketamine and levobupivacaine compared to the placebo are also described by one author in each case. 

In seven of the RCTs the wound was infiltrated prior to wound closure, while in six of them preincisional infiltration was preferred. Three RCTs compare the preincisional to prior-to-skin-closure infiltration. In two studies, infiltration took place after wound closure; the one was applied through a mini vac system to the thyroid bed and the other subcutaneously. 

## 4. Discussion

Except for one, the majority of authors agree that thyroidectomy wound infiltration consists of an easy-to-reproduce technique operated by the surgeon himself, while it elongates the time frame until rescue analgesia. Both the efficacy and the duration of the method depend on surgical incision length, as well as the topical agent used [3,4,5,6,7,8,9,10,11,12,13,14,15,16,17,18,19,20]. Miu et al., could not prove any significant analgesic benefit with ropivacaine infiltration at the end of thyroid surgery [11].

### 4.1. Bupivacaine Infiltration

By using local infiltration of bupivacaine 0.5% prior to skin closure Gozal et al., and Sellami et al., try to investigate the pain management of patients undergoing thyroidectomy. They conclude that there are significantly lower VAS scores, as well as a reduction in morphine consumption during the first post-operative day [3,16]. Along with the difference in infiltrating after wound closure through a mini vac drain, Teksoz et al., also show that the VAS scores of the two groups were significantly lower in the bupivacaine-administered group at 30 min until 8 h post-operative. However, on the first postoperative day the VAS scores were at similar levels in both groups. Rescue analgesia with IV paracetamol was also lower in the bupivacaine group [13].

Dumlu et al., on the other hand, compare two ways for administrating bupivacaine: paratracheal infiltration and subcutaneous infiltration [12]. When assessing the VAS score at 1, 4, and 12 h following the thyroidectomy operation, only the paratracheal infiltration with bupivacaine was significantly lower than that of the control group patients. Demands for rescue analgesia, though, were lowest in subcutaneous infiltration with the bupivacaine group [12]. It seems that infiltration of local anesthetics for thyroidectomy pain control should involve not only the trachea, muscles, and subcutaneous tissues, but also the paratracheal areas, where major dissection takes place. These findings were proposed also by Lacoste et al., since in their RCT, bupivacaine could not provide lower supplementary analgesic requirements, lower VAS scores, or higher patient satisfaction rate than sublingual buprenorphine [4].

Two papers with conflicting results utilize preincisional infiltration with bupivacaine [7,15]. Bagul et al., claim that preincisional subcuticular infiltration with bupivacaine 0.5% is an effective method of pain management in the time-frames tested, while it reduces opioid and analgesic demand [7]. However, Mismar et al., found no significant differences at any time point, whether bupivacaine was administered with adrenaline or not [15]. It is quite noteworthy that in this study the control group requested the lowest average amount of pethidine per patient; this is accredited to the fact that post-thyroidectomy pain is not as severe as expected [15]. 

When comparing preincisional to preclosure bupivacaine infiltration, the superiority of the former in terms of lower VAS scores in the early postoperative period, less analgesic consumption in both the intraoperative and postoperative periods, postponement of first analgesic requirement, and higher patient satisfaction are reported. According to AlSaif et al., the preincional group requested fewer analgesics, while the time until the first analgesic request was significantly delayed. However, the total consumption of pethidine and the pain score revealed no differences. The authors highlight the need for a larger sample size [6]. Similarly, Ekinci et al., although reporting a significant difference in VAS scores and analgesic consumption of the two groups, wonder whether these results are clinically significant since no numerical difference is shown [14].

### 4.2. Ropivacaine Infilration

Ropivacaine infiltration has been studied in three RCTs. However, different protocols were applied. An end-of-surgery wound infiltration with 15 mL of ropivacaine 2% provided a significantly lower VAS score, lower morphine requirements and shorter PACU length of stay. This difference was not observed in the surgical ward, though [9].

Emphasizing multimodal regimens, a recent RCT compares combined 5 mL ropivacaine 0.5% wound infiltration and intravenous flurbiprofen axetil to a single dosage of tramadol [18]. The authors conclude that the maximum postoperative pain scores in the multimodal group were relatively lower than those of the control group at the same time points, while multimodal analgesia seemed to postpone the occurrence of maximum postoperative pain and achieved pain relief during the early postoperative period (2–8 h postoperatively) [18]. On the other hand, Miu et al., could not prove any significant analgesic benefit when ropivacaine was applied at the end of thyroid surgery above the platysma muscle. VAS scores, opioid requirements and complication rate were similar in each study group [11]. 

The authors, trying to explain their negative result, suppose that locoregional techniques may not be appropriate in thyroid surgery, since the postoperative pain is associated not only with the surgical wound but also with the manipulation of tissues, with endotracheal intubation as well as the neck hyperextension position. Possibly, wound infiltration without the recognition of a specific anatomical landmark cannot cover each pain source [11]. When administering the combination of ropivacaine wound infiltration plus intravenous flurbiprofen axetil, both local and systemic analgesia is offered. A better analgesic result is more possible in the multimodal cases where coverage is offered.

### 4.3. Lidocaine Infiltration

Two studies referring to lidocaine infiltration of the thyroidectomy wound were detected. The first compared the preincisional and preclosure infiltration groups [5], while Gau et compared between 5 and 10 mL of lidocaine as a part of a multimodal regimen 20. Chou et al., showed a significantly lower VAS score and lower demands in supplementary analgesia in the preincisional and preclosure group compared to the placebo group. For these two groups, a trend towards delayed requirements for analgesics is also reported. TSH, cortisol, renin and ACTH plasma levels, although elevated, show no difference between the groups [5]. The efficacy of lidocaine as a part of a multimodal protocol though, was also suggested by Gau et al. The authors also showed that the higher volume of lidocaine was superior in terms of pain score on the day of operation and even during the first postoperative week [20].

### 4.4. Ketamine Infiltration

Another RCT compares ketamine wound infiltration with intramuscularly injected ketamine and a placebo. According to the results, ketamine, regardless of the given pathway, and in the immediate postoperative hours, achieved significantly lower VAS scores at rest and at movement than the placebo. Significance could not be proved for the next few hours. No significance was observed between the infiltration and the intramuscular group. The time until rescue analgesia, as well as total opioid consumption, was significantly reduced in the infiltration group compared with the other two groups and in the intramuscularly injected group compared with the placebo [17].

### 4.5. NSAID Infiltration

Lornoxicam as an infiltrative agent in the thyroidectomy wound before skin closure seems not to reduce postoperative analgesic consumption. Kilbaş et al., show that pain scores during the first 24 postoperative hours, although slightly lower in the infiltration group than in the placebo group, are not significantly lower. Lornoxicam by PCA is used as rescue analgesia. Mean consumption follows the same pattern as the VAS score [10]. Regardless of the infiltrative efficacy, the authors suggest that lornoxicam can be an alternative to opioids when administered by PCA for the treatment of moderate-to-severe pain.

### 4.6. Other RCTs

Two studies compare different agents. Karamanlioglu et al., stated that the postoperative combined infiltration of ropivacaine 0.75% plus lornoxicam 8 mg reduced postoperative pain scores during the first 12 postoperative hours, compared with saline [8]. The combination group required less total opioid consumption in the first 24 postoperative hours, while the time to the first analgesic was significantly longer than in the placebo group. Ropivacaine 0.75% or lornoxicam alone could not reach the effectiveness of their combination, though [8]. Worth highlighting is the fact that lornoxicam alone was superior in pain control at 6 h and 8 h post-operatively as compared to the placebo. 

Loh et al., based on the above, decided to study the preincisional infiltration of lornoxicam alone compared to bupivacaine [19]. Diclofenac infiltration showed a trend towards a lower mean pain score at all time intervals. Although statistical significance was only seen at 2 h postoperative at rest, on movement the statistically significant differences were seen at postoperative 1 h, 2 h, 4 h, 6 h and 12 h. Significance was also noted for rescue analgesia with tramadol in the first 24 h post-operative, and was lower in the diclofenac group than in the bupivacaine group [19]. 

## 5. Conclusions

The pain after thyroid surgery is not considered by many physician, misjudging the short incision. However, thyroid surgery induces brief postoperative pain caused by several mechanisms. Cervicotomy, orotracheal intubation, and the cervical hyperextension position are responsible for the postoperative discomfort, neck pain, and shoulder pain. Postsurgical anesthesia remains critical, even in such cases. In the era of multimodal anesthesia we aimed to discuss the role of local infiltration in thyroidectomy. In most cases, it seems that, regardless of the infiltrative agent, the technique has potential. However, the clear superiority of one agent over the other has not been established due to the lack of trials and the relatively limited sample size, as well as the variety of protocols for the existing agent. Perhaps, utilizing larger sample sizes, a variety of local and systemic controls, and infiltrating multiple agents could form part of a future work. Authors should report in detail their methodological plan, the infiltrative technique and the doses applied, as well as key outcomes including analgesic consumption 24 h postoperatively, mean pain scores, time-to-first-analgesic use, and rate of adverse effects. Moreover, concomitant analysis of blood parameters such as IL-6, TNF-a, and cortisol could also help to objectify pain perception.

## Figures and Tables

**Table 1:** Summary of the reported trials.

Year/Author	Study Design/Randomization	Preparation Investigated (Dose)	Time Given	Study Group	Opiod Consumption	Total Analgesic Requirements
2023/Gau [20]	Retrospective observational research	5 and 10 mL of 1% lidocaine plus epinephrine	preincisional infiltration	Group I: 5ml of 1% lidocaine and epinephrine wound infiltration. Group II: received 10 mL of 1% lidocaine and epinephrine wound infiltration. 40 mg intravenous parecoxib before skin excision was given to all patients.	NR	NR
2020/Loh [19]	Prospective double-blinded RCT/sealed envelope method	diclofenac vs. bupivacaine	preincisional	Group I: diclofenac infiltration. Group II: bupivacaine infiltration.	Tramadol Group I: 36.2 mg ± 45.1 Group II: 13.8 mg ± 24.9 *p* = 0.01	Paracetamol Group I: 3063.8 ± 894.5 Group II: 2957.5 ± 1020.6 *p* 0.59 Celebrex Group I: 242.6 ± 171.6 Group II: 183.2 ± 190.1 *p* 0.11
2019/Li [18]	Prospective double-blinded RCT/Computer-generated	5 mL of 0.5% ropivacaine	preincisional	Group 1: 5 mL of 0.5% ropivacaine mixed with epinephrine. Group II: no wound infiltration. At 20 min before the end of surgery, Group I received 100 mg flurbiprofen axetil while Group II received 100 mg tramadol intravenously, followed by injection of 12.5 mg dolasetron in both groups to prevent PONV.	NR	Additional rescue analgesics were not usually required, and the need for additional rescue analgesics did not significantly differ between the groups.
2018/Abd El-Rahman [17]	Prospective double-blinded RCT/Computer-generated	local ketamine 1 mg/kg	prior to skin closure	Group I: normal saline infiltration. Group II: ketamine wound infiltration. Group III: intramuscular ketamine infiltration.	Total morphine consumption was reduced in Group I compared with that in Groups II and III, and in Group II compared with that in Group III (*p* = 0.000).	NR
2018/Sellami [16]	Prospective double-blinded RCT/Computer-generated	10 mL bupivacaine 0.5%	after wound closure	Group I: saline infiltration. Group II: bupivacaine infiltration.	Morphine Group I: 8 mg Group II: 0 mg *p* < 0.001	Nefopam Group I: 17 patients Group II: 5 patients *p* = 0.001
2018/Mismar [15]	Prospective double-blinded RCT/sealed envelope method	bupivacaine 0.5%	preincisional	Group I: bupivacaine, infiltration. Group II: bupivacaine 0.5% with adrenaline infiltration. Group III: no infiltration. Tramadol 50 mg was administered orally every 12 h and paracetamol 1 g was administered orally every 8 h to all patients.	Total pethidine amount per group Group I: 950 Group II: 1200 Group III: 1050	NR
2017/Ekinci [14]	Prospective double-blinded RCT/sealed envelope method	levobupivacaine 0.25%	1. Preincisional 2. After incision	Group I: levobupivacaine infiltration before skin incision. Group II: levobupivacaine infiltration after incsion.	Tramadol Group I: 72.4 ± 24.5 Group II: 91.5 ± 36.8 *p* 0.019	NR
2016/Teksoz [13]	Prospective double-blinded RCT/Computer-generated	bupivacaine 0.5%	after skin closure via mini-vac system	Group I: bupivacaine infiltration. Group II: NaCl (0.9%) infiltration.	NR	Paracetamol Group I: 13 patients Group II: 22 patients *p* 0.005 Each patient in both groups needed no more than one dose.
2016/Dumlu [12]	Prospective double-blinded RCT/NR	bupivacaine 0.5%	prior to skin closure	Group I: no intervention. Group II: paratracheal bupivacaine infiltration. Group III: subcutaneous bupivacaine infiltration	NR	Diclofenac sodium Group I: 26 (86.7%) Group II: 25 (83.0%) Group III: 20 (73.3%) *p* 0.049
2016/Miu [11]	Prospective double-blinded RCT/NR	10 mL ropivacaine 0.75	Prior to skin closure	Group I: sodium chloride infiltration. Group II: ropivacaine infiltration. Patients received 1 g of IV acetaminophen every 6 h during the first 24 h.	Total opioid dose Group I: 64 Group II: 69 *p* 0.20	NR
2015/Kilbaş [10]	Prospective double-blinded RCT/NR	lornoxicam	Prior to skin closure	Group I: lornoxicam infiltration. Group II: saline infiltration. Postoperative analgesia was maintained by intravenous infusion of lornoxicam.	NR	Lornoxicam Group I: 8.87 ± 1.87 Group II: 10.33 ± 1.25 *p* 0.416
2007/Motamed [9]	Prospective double-blinded RCT/Computer-generated	15 mL ropivacaine 2%	prior to skin closure	Group I: ropivacaine infiltration. Group II: saline infiltration. 1 gr paracetamol was injected in all patients.	Amount of morphine in PACU Group I: 6 ± 3 Group II: 13 ± 6 *p* 0.05 Additional 5mg morphine once Group I: 2 patients Group II: 3 patients.	NR
2005/Karamanlioglu [8]	Prospective double-blinded RCT/Computer-generated	10 mL ropivacaine 0.75%, 2 mL lornoxicam (8 mg)	Prior to skin closure	Group S: saline infiltration. Group R: ropivacaine plus 2 mL saline. Group L: lornoxicam (8 mg) plus 10 mL saline. Group RL: ropivacaine plus lornoxicam.	NR	Meperidine Group S: 34.0 ± 33.0 Group R: Group L: Group RL: 78.0 ± 29.8 Total meperidine consumption was significantly less in ropivacaine plus lornoxicam (RL) group when compared with saline group. *p* 0.001
2005/Bagul [7]	Prospective single-blinded RCT/Computer-generated	10 mL bupivacaine 0.5%	preincisional	Group I: infiltration. Group II: no infiltration.	NR	NR
2004/AlSaif [6]	Prospective double-blinded RCT/NR	10 mL bupivacaine 0.5%	preincisional vs. prior to skin closure	Group I: bupivacaine infiltration five minutes before skin incision, and 10 mL of 0.9 saline infiltration at the end of surgery. Group II: saline infiltration before surgical incision, and bupivacaine infiltration at the end of surgery. Group III: 10 mL of 0.9% saline both before and at the end of the surgery.	Pethidine Group I: 125.3 ± 42.5 Group II: 143.3 ± 44.2 Group III: 191.6 ± 54.6 The difference between group C and other two groups A and B was statistically significant (*p* < 0.05).	
1999/Chou [5]	Prospective double-blinded RCT/Computer-generated	10 mL lidocaine 1%	preincisional vs. prior to skin closure	Group I: infiltration prior to skin incision. Group II: infiltration.after surgery. Group III: saline prior to skin Incision.	Pethidine Group I: 640 Group II: 200 Group III: 250 *p* > 0.05	NR
1997/Lacoste [4]	Prospective double-blinded RCT/Computer-based	10 mL bupivacaine 0.25%	prior to skin closure	Group I: oral controlled-release morphine. Group II: sublingual buprenorphine (0.2 mg). Group III: bupivacaine solution.	Morphine Group I: 0.41 + 0.59 Group II: 0.17 + 0.37 Group III: 0.34 ± 0.47 *p* < 0.05	Paracetamol Group I: 0.54 + 0.53 Group II: 0.36 + 0.53 Group III: 0.45 + 0.49 *p* < 0.05
1994/Gozal [3]	Prospective single-blinded RCT/NR	10 mL bupivacaine 0.5%	prior to skin closure	Group I: infiltration. Group II: no infiltration.	Opiod units Group I: 7 Group II: 30 *p* < 0.05	NR

RCT: randomized controlled trial, NR: not referred.

## Data Availability

No new data were created.
